# The Relationship between Plants Used to Sustain Finches (Fringillidae) and Uses for Human Medicine in Southeast Spain

**DOI:** 10.1155/2012/360913

**Published:** 2012-04-30

**Authors:** Antonio Belda, Victoriano Peiró, Eduardo Seva

**Affiliations:** ^1^Departamento Ciencias de la Tierra y del Medio Ambiente, Universidad de Alicante, Campus San Vicente, Apartado 99, 03080 Alicante, Spain; ^2^Departamento de Ecología, Universidad de Alicante, Campus San Vicente, Apartado 99, 03080 Alicante, Spain; ^3^IMEM, Universidad de Alicante, Campus San Vicente, Apartado 99, 03080 Alicante, Spain

## Abstract

We analyzed plants that are traditionally used by wild bird hunters and breeders to capture and promote captive breeding of *Fringillidae* (finches or songbirds) in the province of Alicante, Spain. The majority of plants used in songbird breeding have medicinal properties in traditional human medicine (48 different uses); thus, another main goal was to show their relationships with human medical uses. We compiled a list of 97 plant species from 31 botanical families that are used to attract finches and identified 11 different use categories for these plants in finch keeping. The most common uses were for trapping birds and as a source of food for birds in captivity. *Cannabis sativa* has the greatest cultural importance index (CI = 1.158), and *Phalaris canariensis* (annual canary grass or alpist) was the most common species used to attract Fringillidae and was used by all informants (*n* = 158). Most of the 97 species are wild plants and mainly belong to the families Compositae, Gramineae, Cruciferae, and Rosaceae and also have medicinal properties for humans. In the study area, the intensification of agriculture and abandonment of traditional management practices have caused the population of many songbirds to decline, as well as the loss of popular ethnographic knowledge.

## 1. Introduction

Throughout the ages, the human race has used plants for various purposes [1], particularly those that are accessible. In the Iberian Peninsula, several studies have been developed on medicinal plants [2–8] and edible flora [9, 10], as well as some general ethnobotanical studies [11–15], and others about the importance of home gardens and cultivated areas in the evolution of useful flora [16]. However, few studies have described the use of plants in ethnoveterinary medicine [17, 18], or in attracting and maintaining birds of the Fringillidae family in captivity [19, 20]. Plants have been used in traditional medicine for several thousands of years to treat and cure diseases in domestic animals and human populations, especially native ones [21, 22]. Furthermore, in nature, wild birds use particular plant species, which possess insecticidal and bactericidal properties, to build their nests. This practice creates optimal conditions for egg laying and incubation [23].

The ecological knowledge of local traditional uses that depend on the dynamics of natural resources has been reflected in numerous studies [24–26], considering the ecological knowledge of local communities of hunters, anglers, and gatherers [27].

The culture of capturing songbirds was introduced to the Iberian Peninsula by the Romans and had its beginnings, as did other forms of hunting, in the absolute necessity of human nutrition. Thus, these birds were traditionally caught as a source of food in Valencia, at least since the 17th century [28]. Today, following old customs and culinary habits, there are still hunters who hunt this group of birds in order to eat them. On the other hand, the term “*pajareros*” describes people who are dedicated to hunting, breeding, or selling birds [29]. Although these birds are not hunted excessively, it is essential to monitor and control illegal methods of hunting Fringillidae and to conserve this family of birds [30].

The capture of birds using a hinged net assembly is a traditional hunting method that is widespread in the province of Alicante and elsewhere in the Iberian Peninsula. These game nets are made with cotton, hemp, or nylon mesh. They are placed on the ground and have a manual activation system; once a bird enters the net, a rope is pulled to trap the bird inside the net (see Photographic annex). The nets used since the middle ages to capture several species of Fringillidae intended for use as pets are well known among the inhabitants of this zone [31, 32]. These birds are relatively easy to maintain and rear in captivity, and it is easy to train them to participate in singing competitions. Thus, at present, the capture of five species of birds (*Serinus serinus*, “verdecillo”, *Carduelis carduelis, *“jilguero”, *Carduelis chloris*, “verderón”, *Carduelis cannabina*, “pardillo,” and *Fringilla coelebs*, “pinzón”) is authorized and regulated by law (Council Directive 79/409/EEC and national Laws 4/1989, 62/2006, and 13/2004). What is more, it is an important cultural movement around the Mediterranean Basin [33]. The current trend is to increase breeding in captivity and reduce the capture of wild birds. Therefore, it is important to acquire more knowledge about the traditional use of cultivated and wild plants.

The main aim of this paper is to document the cross-cultural comparison between plant uses for songbirds and humans in Mediterranean environments, relating an ethnoveterinary field study and its eventual link to folk therapies for humans, in order to preserve ethnological knowledge on European folk health. With this purpose in mind, the information on plant uses for songbirds (capturing, feeding, and breeding) gathered here was collected during fieldwork and complemented with ethnobotanical references. Finally, we would like to contribute to the dissemination of results within the scientific community in order to open a door to research in other disciplines.

## 2. Materials and Methods

### 2.1. Study Area

**Figure 1:**
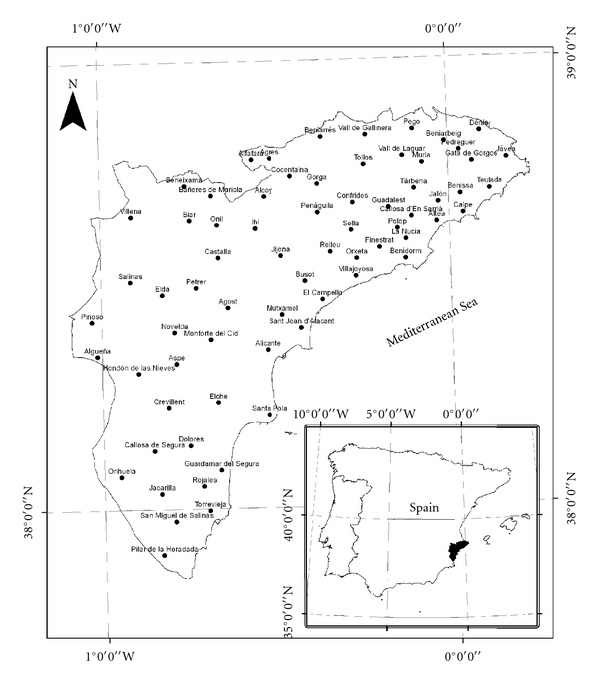
Map showing the location of the province of Alicante (Spain). Dots represent the localities that were prospected (*n* = 69).

The province of Alicante is located in the southeast region of Spain, in the southern part of Valencia. It is geographically located between the coordinates 38°30′N and 0°50′E (Figure 1). The total area occupied by the province is 5,863 km^2^, it has a population of 1,783,555 inhabitants, and there are 141 localities. The province has a very mountainous and rugged relief, except for some river valleys. Thus, approximately 60% of the study area is located between elevations of 200 and 1,500 m above sea level.

Due to its geographical location, the province of Alicante has a typical Mediterranean climate with mild temperatures. Thus, the average temperatures are between 6.2° and 16.8° in the coldest month (January) and 20.4° and 30.6° in the hottest (August), with an annual mean of 17.8°. The average annual rainfall is 336 mm, concentrated in spring and autumn, and there is a prominent dry period in summer. However, there are some climatic differences between the coast and the interior of the province, due to its topography [34, 35]. The plant species in the province of Alicante include sclerophyllous shrubs and trees, which are adapted to Mediterranean stress conditions. Local flora, consisting of evergreen, coriaceous, glabrous, and aromatic plants, is adapted to conserve water for much of the year. Some qualities are common to many of these plants, including resistance to drought, adaptations to heat, and low tolerance to low temperatures. These bioclimatic and biogeographical conditions favour the development of rare, endemic, and endangered species [35, 36]. Considering its bioclimatic and biogeographical conditions, the province of Alicante may potentially give rise to vegetation that can be divided into three main types: evergreen oak forest (*Rubio longifolia-Quercetum rotundifoliae*), ash-maple forest (*Fraxino orni-Aceretum granatensis*), and spiny maquis (*Chamaerops humilis—Rhamnus lycioides*) [37].

### 2.2. Ethnology

A total of 69 localities were prospected with oral interviews in all regions of the Alicante province (*El Comptat, L'Alcoia, Alt Vinalopo, Vinalopo Mitja, Marina Baixa, Marina Alta, L'Alacanti, Baix Vinalopo*, and* Baix Segura-Vega Baixa*) (Figure 1). Vernacular names of plant species traditionally used were obtained in the field by interviews with the local population. Ethnological information was based primarily on semistructured interviews, in which we gathered information. This ranged from the different plant species used to attract and maintain songbirds, the season of plant collection, traditional uses of the plant species collected, the composition of commercial mixtures used to feed captive birds, and folk remedies used to cure songbird illnesses, to the environmental problems faced by the community.

People with a specific profile were selected in order to obtain high-quality and reliable information. People interviewed were older (50–85 years old), living in a rural environment and from a variety of socioeconomical strata, who had captured and bred birds throughout their lives. We wanted to emphasize the ethnobotanical importance of local variations of plant names and the different applications of these species. We conducted 158 oral interviews; 95.57% (*n* = 151) of the informants were male and 4.43% (*n* = 7) female, and the mean age was 56.7 years. In 48 municipalities, inhabitants speak Valencian (variant of Catalan), and Castilian (standard Spanish) is spoken in the others.

Numerous folk botanical references were examined [38–41], including a variety of local books [35], magazines [7], and festivals, to obtain information on remedies for animal illnesses. Even though the information included in our analysis arose from an array of different spoken and written sources in the study area, the semistructured interviews revealed many important issues previously unidentified [20].

A digital sound recorder was used to record interviews and to create an audio record of the information. In addition, a photographical archive, with photographs of each of the species referred to by the informants, was constructed and deposited in the Ecology Department Archive of Alicante University.

The information gathered in interviews was further verified by field observations with the stakeholders. This kind of investigation, in sociological terms, is called “participant observation” [42]. In this process, hunters were observed while they prepared for the hunt and during hunting, and their recreational activities were documented. In these field samplings, we also identified species of plants currently used by bird breeders and the techniques used to catch birds.

Plants were collected from various parts of the study area and were identified in the laboratory, using dichotomous keys [43] and registered at the ABH (Herbarium of Alicante University). We used Excel 2003 to perform a simple statistical analysis of the data collected; specifically, we calculated the relative frequency of citation (RFC) [8] at which each species of plant was used to attract birds during hunting and to maintain birds in captivity (in Table 1). Moreover, we calculated a cultural importance index (CI) where each addend is a measure of the relative importance of each plant use [8].

Finally, we related the use of these plants for wild finches with their potential human medical use, by using some important sources of reference for ethnobotanical and alternative medicine for Mediterranean environments in the southeastern part of Spain [7, 35, 39–41, 44]. Plant uses have been grouped according to cures for different ailments [18].

## 3. Results

We collected 97 species of plants and another variety of one of these species, belonging to 31 botanical families, which are used for different purposes. We present the scientific names of these plant species, voucher register, the family to which they belong, their main uses in finches, relative frequency of citation, cultural importance, whether wild or cultivated types were used, and their medical properties for humans (Table 1).

Compositae, Gramineae, Cruciferae, and Rosaceae are the families most represented among the plants used to catch and promote breeding of songbirds. In this study, all the species of birds showed a preference for wild species of plants. 

### 3.1. Uses in Finches

The most important plant species used by bird breeders are *Phalaris canariensis, Cannabis sativa, Stipa tenacissima, Diplotaxis erucoides,* and *Brassica napus,* representing more than 90% of relative citation frequency (RFC). Among the species with the greatest cultural importance, two species with values higher than 1 for the CI index are striking: *Cannabis sativa* (CI = 1.158) and *Phalaris canariensis* (CI = 1). In contrast, the lowest CI are in *Citrus limon* (CI = 0.025), *Chelidonium majus, Cicer arietinum, Ocimum basilicum* (CI = 0.038), *Allium sativum*, and *Olea europaea* (CI = 0.051). 

**Figure 2:**
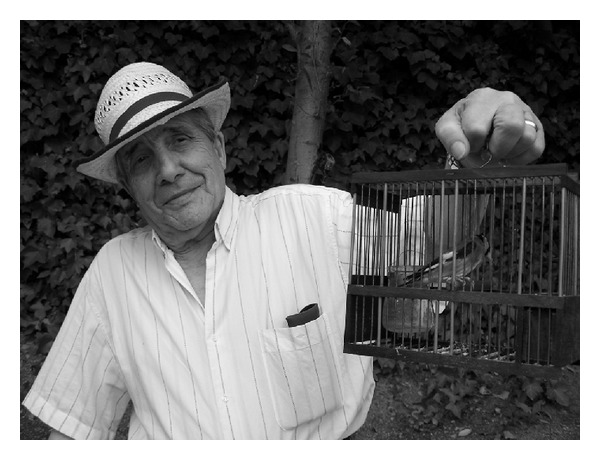
Local bird breeder.

**Figure 3:**
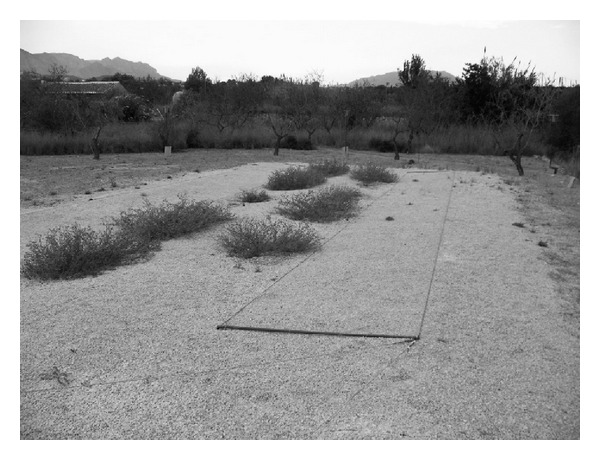
Traditional hunting method using nets.

**Figure 4:**
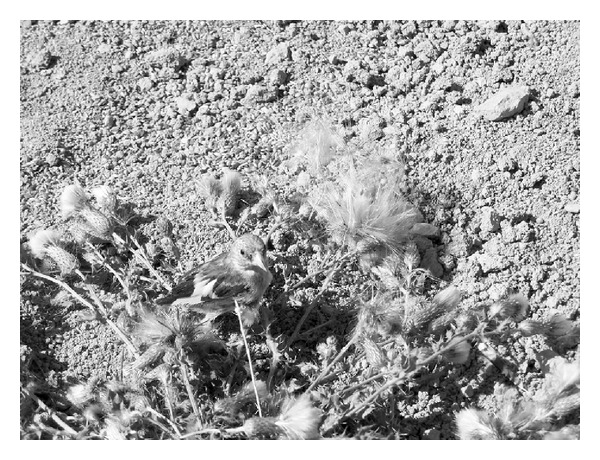
Goldfinch claim in *Centaurea aspera. *

Most of the plant species (24.75%) identified were placed inside nets to attract and capture wild birds in the field (Figure 2). Thus, once birds have entered the nets, the hunter pulls a rope, and the birds are trapped (Figures 3 and 4). The stems of some plants (e.g., Lygeum spartum, Olea europaea and Stipa tenacissima) are spread with an adhesive substance called birdlime (“envisque” or “liga” in local Spanish), obtained from a mixture of resins (e.g., resin from *Pinus halepensis* and *Pinus pinea*), olive oil (from *Olea europaea*), and some plants (e.g., *Andryala ragusina, Chondrilla juncea,* and *Euphorbia characias*). Birds that land on these stems while frequenting feeders or watering points are captured in this way. Catching tools include plants that are used to construct hunter refuges (e.g., *Arundo donax, Phragmites australis,* and *Viscum album*) or decoys that are used to attract other birds to the nets (e.g., *Brachypodium retusum* and *Hyparrhenia hirta*). Capture nets must blend in with the terrain conditions; therefore, they are dyed a matte colour that is as close as possible to the surrounding environment. Hunters use an infusion of certain plants (e.g., *Punica granatum, Pinus halepensis,* and *Pinus pinea*) to produce these dyes. 

Furthermore, many of the species were used to produce the seeds and wild vegetables (18.81%) used to feed birds in captivity. Plants that facilitate breeding include the ones that are used by birds in captivity to build nests (e.g., *Agave americana, Cannabis sativa, Chamaerops humilis, Paronychia argentea*, and *Phoenix dactylifera*), feed their offspring (e.g., *Brassica oleracea* var. *italica*), and stimulate mating (e.g., *Urtica dioica* and *Urtica urens*). Breeders used the fruits and roots of some plants (e.g., *Daucus carota, Fragaria vesca*, and *Rubus ulmifolius*) to enhance the natural red factor in some species of birds, providing natural pigments, particularly in *Carduelis cannabina* and *Carduelis carduelis*. Currently, the cages are made principally from metal or synthetic materials; however, informants can identify the specific natural materials that are used to be used to build cages and cage accessories (e.g., *Arundo donax, Daphne gnidium, Phragmites australis*, and *Nerium oleander*). 

Birds in captivity may suffer from certain diseases, and breeders often try to cure these birds by using natural, plant-based remedies. Thus, there are some vulnerary plants (e.g., *Chelidonium majus* and *Rosa agrestis*) and others that stop haemorrhages (e.g., ash of *Nicotiana tabacum*). Some species have antibacterial properties (e.g., *Cicer arietinum,* vinegar of *Malus domestica,* and *Citrus limon*), or they promote moulting (e.g., *Lavandula latifolia*), have disinfectant functions to eliminate microbes (e.g., *Pistacia lentiscus*), or can host beneficial probiotic bacteria or tonic (e.g., vinegar of *Malus domestica*). Some plants have been used as vermifuge, placed in the breeding carrier, in order to expel parasites (e.g., worms) from the intestines, such as mites (especially *Syringophilus* sp., *Dermoglyphus* sp., and *Dermanyssus* sp.) and lice (*Menacanthus* sp. and *Goniocotes* sp.) that affect this group of birds. Leafy vegetables are used as a laxative treatment, the juice of *Urtica urens* to prevent anaemia, and *Cicer arietinum* is used to stop diarrhoea. To sum up, we show the number of species that are used with specific bird veterinarian uses in Table 2.

### 3.2. Human Medicine Uses

According to the ethnobotanical references consulted, we found 57 plants used in finches that have medical properties in humans. These species are used to cure some ailments related to each pathological group (Table 2). Thus, 48 human uses have been detected in the 97 plant species collected in the study area. *Silybum marianum* (15), *Olea europaea* (12), and *Centaurea aspera* (12) are the species with greater therapeutic uses. We found that 48 uses were related to medical properties: alteration of blood pressure (*n* = 9), haemorrhoids (*n* = 3), depurative (*n* = 9), anxiety (*n* = 9), diarrhoea (*n* = 9), heartburn (*n* = 2), indigestion (*n* = 8), liver disease (*n* = 9), loss of appetite (*n* = 19), constipation(*n* = 11), helminthiasis (*n* = 8), cough (*n* = 7), cold (*n* = 16), respiratory problems (*n* = 8), hyperglycemia (*n* = 9), anaemia (*n* = 2), hypercholesterolemia (*n* = 1), retention of liquids (*n* = 16), undefined symptom (tonic) (*n* = 7), gout (*n* = 3), rheumatism (*n* = 5), inflammation of bones or joints (*n* = 11), undefined symptom (analgesic) (*n* = 4), injury (*n* = 13), burns (*n* = 4), kidney stones (*n* = 2), menstruation (*n* = 5), lack of breast milk secretion (*n* = 2), ischocholia (*n* = 6), chilblains (*n* = 3), pimples (*n* = 5), skin diseases (*n* = 3), eczema (*n* = 5), skin fungus (*n* = 1), rubefaction (*n* = 1), calluses and skin hardness (*n* = 3), warts (*n* = 11), bacteria (*n* = 2), microbes (*n* = 5), headache (*n* = 2), inflammation (*n* = 11), fever (*n* = 3), alopecia (*n* = 1), flushing (refreshing) (*n* = 3), alcoholism (*n* = 1), toothache (*n* = 8), mineral deficiency (*n* = 1), and eye infection (*n* = 2). 

We only found three vulnerary plants for finches; however, there are 13 species of the total used for this use in humans. There are three antibacterial plants in birds, while in humans we found two different species (*Portulaca oleracea* and *Centaurea aspera*). One plant is disinfectant for finches, while in humans there are 5 antiseptics to eliminate microbes (*Foeniculum vulgare, Centaurea aspera, Pinus halepensis, Lavandula latifolia, *and* Rubus ulmifolius*) and fungal species (*Centaurea aspera*). Twenty eight species are used as a laxative treatment in birds, whereas only eleven have the same medical use for humans. Conversely, we found no plants that are probiotic or that stop bleeding in humans. 

## 4. Discussion

Traditionally, nutritive uses [45] and curative applications [46] of ethnobotanical knowledge have been linked to women. They have demonstrated a high knowledge of both wild and cultivated species [47, 48], especially in rural areas [7]. In contrast, wild bird hunting is traditionally a male-dominated pastime. Therefore, we want to highlight that the stakeholders have high know-how, which reflects their identification of different species and their applications. The names and traditional uses can vary depending on geographical location, as vernacular names serve as intangible heritage. Thus, it is necessary to preserve this heritage and promote educational and awareness programmes [20]. 

The cultural importance index corresponds with an interest in detailing the specific uses of plants that better reflect the cultural aspects of plant utilization. In fact, ethnobotanical publications usually present plant uses in tables or catalogues, where the information is grouped by species, indicating their particular uses and, commonly, the number of informants who mentioned them. This way of grouping is much more reasonable for evaluating the importance of each plant species by its cultural consensus [8]. This additive index takes into account not only the spread of use (number of informants) for each species, but also its versatility, that is, the diversity of its uses [17]. Thus, *Cannabis sativa* and *Phalaris canariensis* have the greatest CI, being the principal commercial seed and, moreover, *Cannabis sativa* has other uses. In contrast, the lowest CI are in plants that are used to cure or have no typical uses and are not used by informants to breed songbirds. 

Various mixes of dried seeds, composed of seeds from different species, both wild and cultivated, are used to feed birds in captivity [49]. Each bird breeder uses the mixture of seeds that he/she deems most appropriate. However, some breeders use leafy vegetables to feed birds and supplement their diet of dried seeds. These plants are used mainly in summer, during the birds' moulting period and as a laxative. Other species not cited in this study, such as *Ilex aquifolium*, *Viscum cruciatum,* or *Onopordum nervosum* [11–13, 50–52], are used to capture birds in other areas. Moreover, some plants also have different veterinary uses in other Mediterranean regions. Thus, some authors show that several species, such as *Stellaria media*, *Avena sativa,* and *Urtica dioica, *are used to increase fertility and egg production in chickens. *Urtica urens* is mixed with feed for hens so that they lay eggs earlier in their lifespan and as a result, the eggshells will be harder. *Cirsium arvense*, *Daphne gnidium*, *Phragmites australis, *and *Linum usitatissimum* are antidiarrhoeal and have been used to favour digestion. *Allium sativum, Daphne gnidium*,* Nerium oleander,* and *Nicotiana tabacum *are useful against parasites on farms, and *Cicer arietinum* is used to facilitate the expulsion of the placenta and for purgation in goats and sheep. *Olea europaea* is used to treat mastitis or to detoxicate, and latex from *Chelidonium majus* and *Pinus halepensis* is used to treat wounds [17, 19, 21, 53]. With these data, we can verify that there is a popular tradition for the use of ethnoveterinary plants in Mediterranean areas. 

Furthermore, some species identified without human medicine use in the study area have them in other Spanish regions [5, 54–57], such as *Avena sativa* (toothache and quitting smoking), *Bituminaria bituminosa *(vulnerary), *Brassica oleracea* var. *italica* (vulnerary, remineralizing, headache, and anthelmintic), *Brassica rapa* (culinary), *Cannabis sativa* (refreshing and relaxing), *Chelidonium majus* (anticholagogue, hepatoprotective, anti-inflammatory, antiseptic, warts, laxative, and vulnerary), *Conyza bonariensis* (digestive), *Helianthus annuus *(febrifuge), *Phagnalon saxatile* (carminative, analgesic, and cholesterol levels), *Phalaris canariensis* (cholesterol), *Scorzonera hispanica* (diuretic, uric acid, and cholesterol), *Senecio vulgaris* (anti-inflammatory and antiseptic), and *Viscum album* (anticatarrhal, antiseptic, antivariolous, parasiticide, salutiferous, and sedative). Other species, such as *Carthamus tinctorius*,* Centaurea mariolensis*,* Centaurea melitensis*,* Guizotia abyssinica*,* Panicum miliaceum*,* Perilla frutescens*,* Setaria italica, *and *Spinacia oleracea, *do not present other applications in humans, according to these references. This may be due to the rarity of these species or that they are not traditionally cultivated species in the area. 

However, some of the species studied in this project are in the group of the top vascular plants in traditional phytotherapy in other regions, such as *Allium sativum* (antinostalgic, anthelmintic, anti-inflammatory/antalgic, antiverrucose, and antibronchitic), *Foeniculum vulgare *(carminative, cold, intestinal anti-inflammatory, laxative, gastralgia, diuretic, and antihalitosis), and *Olea europaea* (antihypertensive, hyperglycemia, hernia, food poisoning, heartburn, warts, cough, erysipelas, sores, psoriasis, burns, hoarseness, baldness, rheumatism, antipyretic, antiseptic, laxative, and antinostalgic) [18, 58]. 

On the other hand, bird populations have declined, mainly due to the abandonment of crops, the use of pesticides, predation of nests, poaching, increased predation due to changes in their natural habitat, uncontrolled development, and in general socioeconomic changes in recent decades [59]. In this aspect, the mechanization of agricultural practices has changed the structure of these agrarian ecosystems, accompanied by a steady degradation and loss of landscape elements with important ecological functions [60]. To preserve bird populations, it is essential to maintain fields active. There are many plants linked to these environments that birds use daily, such as for food or other purposes.

## 5. Conclusions

In conclusion, data obtained in this research are scarcely known and show many details of plants related to songbirds, facilitating access to interesting and novel information. This allows recovery of forgotten uses and traditions, highlighting the utilization of different species to attract and cure birds and their relation to human medicine, and resulting in a very interesting contribution to ethnobotanical bibliography. 

We found that the majority of the plant species related to songbirds were wild, reflecting that the wild bird hunters are aware of this preference and exploit this knowledge of wild flora in their hunting. This demonstrates that informants have great knowledge of the plants used in traditional medicine and finch keeping. Also, the majority of species have medicinal properties that can be used for informants to cure different pathologies.

## Figures and Tables

**Table 1:** Plant species in the study area and their traditional uses in finches and humans. Finch uses: 1, facilitate breeding; 2, attract birds; 3, commercial seed mixes; 4, leafy vegetables; 5, birdliming; 6, material for cages and accessories; 7, catching tools; 8, vermifuge; 9, camouflage of capture nets; 10, provide pigments; 11, cure diseases. Medical human uses: 1, alteration of blood pressure; 2, haemorrhoids; 3, depurative; 4, anxiety; 5, diarrhoea; 6, heartburn; 7, indigestion; 8, liver disease; 9, loss of appetite; 10, constipation; 11, helminthiasis; 12, cough; 13, cold; 14, respiratory problems; 15, hyperglycemia; 16, anaemia; 17, hypercholesterolemia; 18, retention of liquids; 19, undefined symptom (tonic); 20, gout; 21, rheumatism; 22, inflammation of bones or joints; 23, undefined symptom (analgesic); 24, injury; 25, burns; 26, kidney stones; 27, menstruation; 28, lack of breast milk secretion; 29, ischocholia; 30, chilblains; 31, pimples; 32, skin diseases; 33, eczema; 34, skin fungus; 35, rubefaction; 36, calluses and skin hardness; 37, warts; 38, bacteria; 39, microbes; 40, headache; 41, inflammation; 42, fever; 43, alopecia; 44, flushing (refreshing); 45, alcoholism; 46, toothache; 47, mineral deficiency; 48, eye infection. Type: W, wild; C, cultivated. RFC: relative frequency of citation. CI: cultural importance index.

Scientific name	Herbarium voucher (ABH)	Family	Finch uses	RFC	CI	Medical human uses	Type	References
*Agave americana* L.	17879	*Agavaceae*	1	7.59	0.076	—	W	[39]
*Allium sativum *L.	Seen alive	*Alliaceae*	8	5.06	0.051	41, 13, 5, 21, 30	C	[35, 39, 44]
*Amaranthus blitum* L.	3989	*Amaranthaceae*	2,3	25.32	0.285	—	W	[41]
*Anagallis arvensis *L.	22647	*Primulaceae*	4	16.46	0.165	24, 3, 14	W	[40]
*Andryala ragusina *L.	4430	*Compositae*	5	72.15	0.722	31	W	[41]
*Arundo donax* L.	32085	*Gramineae*	6,7	58.23	0.810	1, 48, 12, 22, 18, 3	C	[39]
*Avena sativa *L.	10488	*Gramineae*	3	84.81	0.848	—	W	
*Avena sterilis* L.	1582	*Gramineae*	3	78.48	0.785	—	W	
*Beta vulgaris* L.	10652	*Chenopodiaceae*	4	30.38	0.304	37, 10, 18, 44, 1, 41, 6, 2	C	[41]
*Bituminaria bituminosa* (L.) C. H. Stirt.	50474	*Leguminosae*	2	32.91	0.329	—	W	
*Brachypodium retusum* (Pers.) Beauv.	31248	*Gramineae*	7	67.09	0.671	1	W	[39]
*Brassica napus *L.	39373	*Cruciferae*	3	89.87	0.899	—	C	
*Brassica oleracea* L. subsp. *oleracea *	34847	*Cruciferae*	4	21.52	0.215	24, 28, 45, 41, 21, 18, 10, 48, 4, 7	C	[41]
*Brassica oleracea* L. var. *italica *Plenck	Seen alive	*Cruciferae*	1,4	62.03	0.924	—	C	
*Brassica rapa *L.	7969	*Cruciferae*	3	88.61	0.886	—	C	
*Cannabis sativa* L.	32225	*Cannabaceae*	1,3	92.41	1.158	—	C	
*Capsella bursa-pastoris* (L.) Medicus	47380	*Cruciferae*	4	16.46	0.165	5, 18, 1, 27	W	[40]
*Carthamus tinctorius* L.	3894	*Compositae*	3	10.13	0.101	—	W	
*Centaurea aspera* L.	21338	*Compositae*	2	60.76	0.608	9, 7, 15, 24, 34, 38, 41, 1, 13, 39, 19, 29	W	[7, 35, 40]
*Centaurea calcitrapa* L.	36097	*Compositae*	2	46.84	0.468	15	W	[40]
*Centaurea mariolensis *Rouy	13242	*Compositae*	2	10.13	0.101	—	W	
*Centaurea melitensis *L.	36917	*Compositae*	2	11.39	0.114	—	W	
*Chamaerops humilis *L.	559	*Palmae*	1	13.92	0.139	9	W	[39]
*Chelidonium majus* L.	18328	*Papaveraceae*	11	3.8	0.038	—	C	
*Chondrilla juncea *L.	7142	*Compositae*	5	49.37	0.494	9	W	[39]
*Cicer arietinum* L.	17633	*Leguminosae*	11	3.8	0.038	31, 18, 13	W	[41]
*Cichorium intybus* L.	37547	*Compositae*	3,4	54.43	0.639	18, 19, 10, 9	W	[35, 39, 44]
*Cirsium arvense* (L.) Scop.	35007	*Compositae*	2	8.86	0.089	9, 2	C	[39]
*Cirsium monspessulanum *(L.) Hill	51477	*Compositae*	2	10.13	0.101	—	W	
*Citrus limon *(L.) Burm. Fil.	49856	*Rutaceae*	11	2.53	0.025	24, 5, 3, 18, 13	C	[39]
*Conyza bonariensis *(L.) Cronq.	17943	*Compositae*	2	12.66	0.127	—	C	
*Cynara cardunculus *L.	35991	*Compositae*	2	16.46	0.165	37, 27, 29, 32, 9, 3, 8, 18, 15	W	[7, 35, 41, 44]
*Cynara scolymus* L	31715	*Compositae*	2	44.3	0.443	—	C	
*Daphne gnidium* L.	10830	*Thymelaeaceae*	6,8	41.77	0.481	37, 13, 8, 11, 35	W	[7, 35, 39]
*Daucus carota* L.	33104	*Umbelliferae*	4,10	18.99	0.222	18, 37, 9, 19	W	[35, 39]
*Diplotaxis erucoides* (L.) DC.	47963	*Cruciferae*	2,4	91.14	0.911	—	W	[40]
*Dittrichia viscosa *(L.) Greuter	39371	*Compositae*	2	68.35	0.684	24, 1, 26, 17, 46	W	[39]
*Echinochloa crus-galli* (L.) Beauv.	14692	*Gramineae*	2	6.33	0.063	—	W	
*Eruca vesicaria* (L.) Cav.	41713	*Cruciferae*	4	20.25	0.203	—	W	
*Erucastrum virgatum *C. Presl	4460	*Cruciferae*	2,4	15.19	0.190	—	W	
*Euphorbia characias *L.	7226	*Euphorbiaceae*	5	36.71	0.367	8	C	[39, 44]
*Foeniculum vulgare *Miller	23129	*Umbelliferae*	3	24.05	0.241	18, 33, 10, 14, 48, 13, 9, 8, 39, 28, 7	W	[7, 35, 39, 44]
*Fragaria vesca *L.	52157	*Rosaceae*	4,10	11.39	0.139	13, 30, 20	W	[44]
*Galactites tomentosa Moench*	42051	*Compositae*	2	11.39	0.114	—	C	
*Guizotia abyssinica *(L.f.) Cass.	9666	*Compositae*	3	87.34	0.873	—	W	
*Helianthus annuus *L.	5220	*Compositae*	3	87.34	0.873	—	W	
*Heliotropium europaeum* L.	14672	*Boraginaceae*	2	82.28	0.823	24, 29, 42, 27, 20, 37	W	[35, 41]
*Hyparrhenia hirta* (L.) Staff	41077	*Gramineae*	7	32.91	0.329	—	C	[39]
*Lactuca sativa* L.	Seen alive	*Compositae*	3,4	58.23	0.728	44, 47, 4, 46	C	[41]
*Lactuca serriola* L.	47376	*Compositae*	4	22.78	0.228	10, 4	W	[41]
*Laurus nobilis* L.	43242	*Lauraceae*	8	13.92	0.139	14, 13, 9, 7, 6, 31, 8	C	[35, 39, 44]
*Lavandula latifolia *Medicus	20246	*Labiatae*	4,11	37.97	0.456	19, 7, 39, 24, 21, 18, 33, 14, 41, 5	W	[7, 35, 39, 44]
*Linum usitatissimum* L.	32017	*Linaceae*	3	13.92	0.139	10, 33, 21, 31, 24, 14, 13, 41, 4	W	[35, 39]
*Lobularia maritima *(L.) Desv.	15843	*Cruciferae*	4	35.44	0.354	18, 26, 41, 23, 42	W	[41]
*Lygeum spartum *L.	8128	*Gramineae*	5	45.57	0.456	—	W	
*Malus domestica* (Borkh.) Borkh.	37495	*Rosaceae*	4,11	27.85	0.392	7, 37	W	[40]
*Mantisalca salmantica *(L.) Briq. and Cavill.	5273	*Compositae*	2	13.92	0.139	15	C	[41]
*Nerium oleander* L.	46139	*Apocynaceae*	6,8	30.38	0.411	32	C	[39]
*Nicotiana tabacum* L.	4391	*Solanaceae*	8,11	13.92	0.234	43, 40, 46	W	[39]
*Ocimum basilicum L.*	Seen alive	*Labiatae*	8	3.8	0.038	7, 4, 8, 13, 10, 5	C	[35, 39, 44]
*Olea europaea* L.	17212	*Oleaceae*	5	5.06	0.051	36, 10, 1, 41, 7, 29, 37, 24, 19, 25, 23, 8	W	[7, 35, 40, 44]
*Onopordum acanthium* L.	11328	*Compositae*	2	11.39	0.114	—	W	
*Panicum miliaceum *L.	36589	*Gramineae*	3	68.35	0.063	—	W	
*Papaver rhoeas *L.	37589	*Papaveraceae*	3,4	26.58	0.291	14, 12, 46, 23, 9, 18, 13, 4	C	[7, 35, 39, 44]
*Papaver somniferum* L.	10585	*Papaveraceae*	3,4	17.72	0.203	13, 4, 5, 12, 46	W	[39]
*Paronychia argentea* Lam.	13044	*Caryophyllaceae*	1	6.33	0.063	18, 24, 1	C	[35]
*Perilla frutescens *L.	Seen alive	*Labiatae*	3	60.76	0.608	—	C	
*Phagnalon saxatile* (L.) Cass.	49631	*Compositae*	2	10.13	0.101	—	W	
*Phalaris canariensis *L.	14955	*Gramineae*	3	100	1.000	—	W	
*Phoenix dactylifera *L.	14303	*Palmae*	1	10.13	0.101	9	W	[39]
*Phragmites australis *(Cav.) Steudel	40289	*Gramineae*	6,7	64.56	0.918	—	W	
*Picris echioides *L.	47438	*Compositae*	2	7.59	0.076	—	W	[40]
*Pinus halepensis *Miller	37506	*Pinaceae*	2,5,9	51.9	0.791	14, 13, 41, 19, 37, 24, 39	W	[35, 40, 44]
*Pinus pinea* L.	32768	*Pinaceae*	3,5,9	7.59	0.120	—	C	
*Piptatherum miliaceum* (L.) Coss.	6843	*Gramineae*	3	27.85	0.278	—	C	
*Pistacia lentiscus* L.	10319	*Anacardiaceae*	11	6.33	0.063	37	C	[39]
*Portulaca oleracea *L.	36619	*Portulacaceae*	2,3	72.15	0.759	41, 38, 15, 44, 33, 23, 18, 11, 4, 25	W	[40]
*Punica granatum* L.	46140	*Punicaceae*	9	8.86	0.089	11, 15	W	[40]
*Raphanus sativus* L.	51395	*Cruciferae*	3,4	49.37	0.741	29, 12, 3, 21, 13,	W	[41]
*Rosa agrestis *Savi	51473	*Rosaceae*	11	6.33	0.063	18, 41, 24, 4	W	[35, 44]
*Rubus ulmifolius* Schott	40230	*Rosaceae*	4,10	11.39	0.133	24, 46, 15, 5, 39, 9, 3, 31	W	[7, 35, 39, 44]
*Scolymus hispanicus* L.	20754	*Compositae*	2	40.51	0.405	—	W	
*Scolymus maculatus *L.	20114	*Compositae*	2	10.13	0.101	—	W	
*Scorzonera hispanica* L.	4557	*Compositae*	4	16.46	0.165	—	W	
*Senecio vulgaris* L.	7527	*Compositae*	4	7.59	0.076	—	W	
*Setaria italica* (L.) P. Beauv.	16519	*Gramineae*	3	46.84	0.468	—	C	
*Silybum marianum *(L.) Gaertner	32020	*Compositae*	2	73.42	0.734	21, 8, 9, 3, 36, 37, 27, 29, 32, 15, 1, 42, 12, 13, 40	C	[7, 35, 41, 44]
*Sonchus oleraceus* L.	47365	*Compositae*	4	44.3	0.443	9	W	[39]
*Sonchus tenerrimus* L.	37483	*Compositae*	4	40.51	0.405	9	W	[39]
*Sorghum halepense *(L.) Pers.	3298	*Gramineae*	3	53.16	0.532	46	W	[41]
*Spinacia oleracea* L.	Seen alive	*Chenopodiaceae*	4	43.04	0.430	—	C	
*Stellaria media* (L.) Vill.	10674	*Caryophyllaceae*	4	11.39	0.114	—	W	
*Stipa tenacissima* L.	44375	*Gramineae*	5	92.41	0.924	37	W	[39]
*Taraxacum vulgare* (Lam.) Schrank	1808	*Compositae*	4	49.37	0.494	33, 8	W	[44]
*Thymelaea hirsuta *L.	41262	*Thymelaeaceae*	2	27.85	0.278	11, 10	C	[41]
*Urtica dioica* L.	40147	*Urticaceae*	1,4	26.58	0.316	1, 20, 5, 41, 16, 13	W	[7]
*Urtica urens* L.	33640	*Urticaceae*	1,4,11	34.18	0.424	21, 13, 41, 3, 30, 9, 18, 37, 1, 12, 16	W	[35, 39, 44]
*Viscum album* L.	49508	*Viscaceae*	7	60.76	0.608	—	W	

**Table 2:** Number and frequency of plants used for a specific human use.

Pathologic group	Human use	Medical code	No. of species	Frequency	Bird veterinarian
Circulatory system	Alteration of blood pressure	1	9	9.28	
	Haemorrhoids	2	3	3.09	
	Undefined symptom (depurative)	3	9	9.28	
Mental illness	Anxiety	4	9	9.28	
Digestive system	Diarrhoea	5	9	9.28	1
	Heartburn	6	2	2.06	
	Indigestion	7	8	8.25	1 probiotic
	Liver disease	8	4	4.12	
	Loss of appetite	9	19	19.59	
	Constipation	10	11	11.34	28
	Helminthiasis	11	8	8.25	6
Respiratory system	Cough	12	7	7.22	
	Cold	13	16	16.49	
	Respiratory problems	14	8	8.25	
Metabolism, nutrition, and so forth	Hyperglycemia	15	9	9.28	
	Anaemia	16	2	2.06	1
	Hypercholesterolemia	17	1	1.03	
	Retention of liquids	18	16	16.49	
	Undefined symptom (Tonic)	19	7	7.22	1
	Gout	20	3	3.09	
Bones, joints, and so forth	Rheumatism	21	5	5.15	
	Inflammation	22	1	1.03	
	Undefined symptom (analgesic)	23	4	4.12	
Traumatic injuries and poisoning	Injury	24	13	13.40	3
	Burns	25	4	4.12	
Genital urinary	Kidney stones	26	2	2.06	
	Menstruation	27	5	5.15	
	Lack of breast milk secretion	28	2	2.06	
	Ischocholia	29	6	6.19	
Skin and subcutaneous tissues	Chilblain	30	3	3.09	
	Pimples	31	5	5.15	
	Skin problems	32	3	3.09	
	Eczema	33	5	5.15	
	Skin fungus	34	1	1.03	
	Rubefaction	35	1	1.03	
	Calluses and skin hardness	36	2	2.06	
Infectious and parasitic diseases	Warts	37	11	11.34	
	Bacteria	38	2	2.06	3
	Microbes	39	5	5.15	1
Symptoms, signs, and poorly defined morbid states	Headache	40	2	2.06	
	Inflammation	41	11	11.34	
	Fever	42	3	3.09	
	Alopecia	43	1	1.03	
	Flushing	44	3	3.09	
	Alcoholism	45	1	1.03	
	Toothache	46	8	8.25	
	Mineral deficiency	47	1	1.03	1 molting
Nervous system and sensory organs	Eye infection	48	2	2.06	
